# Curcumin extract addition to bull semen does not adversely affect in vitro fertilization

**DOI:** 10.1186/s13028-026-00890-5

**Published:** 2026-10-29

**Authors:** Aleksandar Cojkic, Olga Sajecka, Ylva Sjunnesson, Jane M. Morrell

**Affiliations:** Department of Clinical Sciences, Swedish University of Agricultural Sciences (SLU), Uppsala, 75007 Sweden

**Keywords:** Bull semen, Curcumin, IVF

## Abstract

**Background:**

Curcumin extract has demonstrated antioxidant and antibacterial properties in bull semen samples at a concentration of 5%. Nevertheless, its impact on fertilization outcomes remains largely unexplored. This study aimed to evaluate if curcumin extract added to bull semen had an effect on in vitro fertilization (IVF) results. Bovine ovaries were collected from an abattoir, cumulus-oocyte complexes (COCs) were aspirated, graded, and randomly assigned to treatment groups before maturation. The COCs were matured for 22 hours and fertilized using semen from one bull with verified fertility. Semen samples were thawed and treated with 5% curcumin extract for 30 minutes at room temperature, and IVF was conducted at a reduced sperm concentration (0.5 × 10^6^/mL) to better assess treatment effects. Cleavage was evaluated, 44 hours post-fertilization, and embryo development was assessed on day 8. Sperm quality parameters were evaluated using Computer-Assisted Sperm Analysis for motility and kinematics, and Flow Cytometry for viability markers, including membrane integrity, mitochondrial membrane potential (MMP), and chromatin integrity.

**Results:**

No statistically significant differences were observed between groups for any IVF outcomes. A trend toward significance was observed for total motility and immotile spermatozoa, with higher motility and lower immotility in the curcumin-treated group compared to controls.

**Conclusion:**

The results demonstrated that curcumin treatment does not seem to have an effect on in vitro fertilization (IVF) outcomes since no statistically significant differences were observed between the curcumin-treated and control groups in cleavage rate, the proportion of oocytes reaching more than two cells, or blastocyst formation on day 8. These findings indicate that, under the experimental conditions of this study, curcumin does not impair the fertilization capacity of bull sperm.

## Background

Reproductive biotechnology has become an essential part of modern livestock farming, largely due to the widespread use of artificial insemination (AI), cryopreservation of semen, and in vitro embryo production [1]. These technologies facilitate the dissemination of superior genetics and the long-term preservation of valuable semen, as well as the efficient production of offspring [1]. However, the processing and cryopreservation of semen can expose spermatozoa to oxidative, osmotic, thermal and mechanical stresses, which may impair membrane integrity, motility, mitochondrial activity and DNA integrity [2]. This can potentially reduce their ability to fertilise oocytes. Consequently, developing safe additives that can protect sperm function during semen preservation is a key objective in reproductive biotechnology [3].

Plant-derived compounds are attracting increasing interest as potential alternatives or complementary additives in reproductive biotechnology, as many of them contain biologically active molecules with antioxidant, anti-inflammatory, antimicrobial, membrane-stabilising or cytoprotective properties [4]. However, their effects are not necessarily uniformly beneficial, and may depend on factors such as the plant source, chemical composition, concentration, solvent or delivery system, species, and reproductive cell type [3, 5, 6]. Curcumin, the major biologically active polyphenolic compound found in *Curcuma longa*, is of particular interest due to its wide range of biological activities. Its antioxidant properties, including its ability to scavenge reactive oxygen species (ROS), modulate antioxidant defence systems and reduce oxidative damage to cellular lipids, proteins and DNA, have been extensively investigated [7, 8]. These properties are particularly relevant to spermatozoa, as oxidative stress contributes considerably to the loss of sperm function during semen storage and cryopreservation. In bull spermatozoa exposed to experimentally induced oxidative stress, curcumin was found to preserve sperm motility, viability, mitochondrial activity and intracellular antioxidant capacity, whilst reducing oxidative damage [9]. Similarly, adding curcumin to semen extenders has improved post-thaw sperm quality in cattle, buffalo, boar and other species, although the magnitude and direction of the response varies with concentration and experimental conditions [10–12]. For instance, curcumin supplementation at suitable concentrations has been shown to enhance motility and improve membrane and acrosome integrity while reducing oxidative and DNA damage in cryopreserved bull spermatozoa [9]. Furthermore, studies on buffalo and boar semen have demonstrated enhanced post-thaw sperm characteristics following curcumin supplementation [11–13].

Another potential application of plant-derived compounds in semen preservation is reducing microbial contamination. Although conventional antibiotics are routinely incorporated into semen extenders to prevent bacterial growth, concerns regarding antimicrobial resistance and persistent bacterial contamination have led to the investigation of alternative antimicrobial substances. While several plant extracts have demonstrated antimicrobial activity in semen, their effects on sperm quality can vary considerably [14].

Importantly, the potential reproductive effects of curcumin go beyond its antioxidant activity in spermatozoa. It has been investigated in several areas of female reproductive biotechnology. In porcine oocytes, adding moderate concentrations of curcumin to the maturation medium improved oocyte maturation and fertilization, it also increased blastocyst formation at a specific concentration [5]. Similarly, recent studies in bovine reproductive models have reported that curcumin supplementation can influence oocyte maturation, antioxidant status, fertilisation and embryo development [6, 15]. In buffalo, curcumin supplementation during embryo culture improved blastocyst development under normal and heat-stress conditions at an appropriate concentration; a higher concentration, however, was less beneficial [16].

Collectively, these findings indicate that curcumin may exert protective effects on both male and female gametes and early embryos, but that these effects depend strongly on concentration and the stage of reproductive development. Studies directly evaluating the impact of natural compound supplementation on fertilisation and early embryonic development remain comparatively limited. Therefore, the aim of the present study was to evaluate if curcumin extract added to bull semen had an effect on in vitro fertilization.

## Material and methods

### Experimental design

**Fig. 1:**
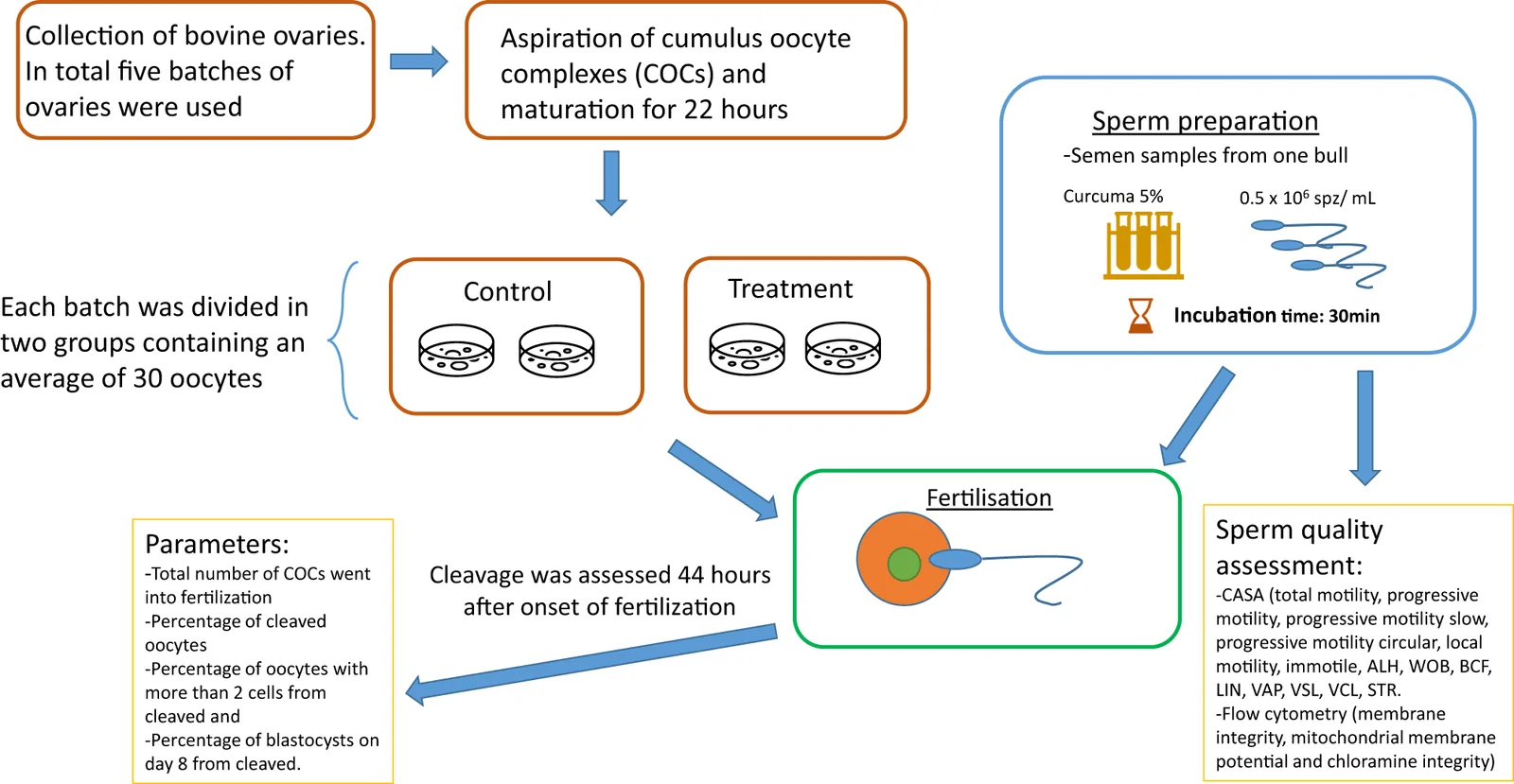
Experimental design

The experimental design is presented in Fig. 1.

In brief, bovine ovaries were collected from an abattoir and transported to the laboratory within 5 hours. Cumulus-oocyte complexes (COCs) were aspirated, selected for quality, and randomly assigned to control or treatment groups. The COCs were matured for 22 hours in Stroebech Media (Stroebech Media ApS, Hundested, Denmark). Five ovary replicates were performed, each split into two groups (~30 oocytes). Fertilization was performed using frozen thawed semen from one proven bull, at a lower concentration than commonly used, to ensure no possibilities for compensatory actions of a high number of spermatozoa. The treatment group received semen pre-treated with 5% curcumin extract for 30 minutes at room temperature after thawing while the control group received the same treatment with only solvent. Cleavage was assessed 44 hours post-fertilization. Parameters evaluated were total number of COCs included, percentage (%) of cleaved oocytes, percentage (%) of oocytes with more than 2 cells (>2 cells), and percentage (%) of blastocysts on day 8 from cleaved oocytes. The concentration of 5% curcumin was chosen since it was previously shown to exhibit antibacterial activity without compromising sperm quality [14].

### Sperm sample

Semen from a single bull of Swedish Red dairy breed with proven field and in vitro fertility was used. Semen samples were thawed in warm water at 37 °C for 12 seconds then divided into two aliquots-treatment and control. Treatment aliquots were incubated at room temperature with curcumin extract at a final concentration of 5% for 30 minutes, while control aliquots were incubated under the same conditions with the corresponding volume of diluted ethanol (solvent control) without curcumin. The concentration of curcumin was selected based on previous findings [14], which demonstrated a significant reduction in bacterial load without detrimental effects on sperm quality parameters after incubation. In detail, the curcumin solution was prepared by dissolving 5 mg of curcumin (Sigma-Aldrich GmbH, Stockholm, Sweden) in 500 µL of 70% ethanol. The solution was subsequently diluted with peptone water (Dilucup) to a final volume of 15 mL, resulting in a curcumin concentration of 667 µg/mL. The preparation was then divided into 5-mL aliquots and centrifuged at 4000 × *g* for 5 min at 22 °C. Following centrifugation, the resulting supernatant was collected and used for semen supplementation. Following treatment, each semen sample was divided into two portions: one portion was used directly for Computer Assisted Sperm Analysis (CASA) and flow cytometric analyses to assess sperm characteristics after curcumin exposure, while the second portion was used for IVF. Thereafter 3.8 mL of Sperm Wash were added to each sample, followed by centrifugation (330 ×g for 5 min). The supernatant was then removed, an additional 3.8 mL of Sperm Wash were added, and the sample was centrifuged once more before adjusting to a concentration of 0.5 × 10^6^ spermatozoa/mL and adding to the oocytes. The sperm concentration was intentionally reduced from the usual sperm concentration of 1 × 10^6^ spermatozoa/mL to better observe the effect of the spermatozoa on the system [17]. For the control group, 500 µL of 70% ethanol (used as the curcumin solvent) was diluted to a final volume of 15 mL with peptone water (Dilucup) to match the solvent conditions of the curcumin-treated samples.

### CASA – computer assisted sperm analyses

Sperm motility was evaluated using the AndroVision® system (Minitüb Abfüll- und Labortechnik GmbH & Co. KG, Tiefenbach, Germany) following a 5-minute incubation at 37 °C. A minimum of four fields, corresponding to approximately 1000 spermatozoa in total, were analysed with the AndroVision® software. The following kinematic parameters were assessed: beat cross frequency (BCF, Hz), linearity (LIN), amplitude of lateral head displacement (ALH, µm), wobble (WOB), velocity of the average path (VAP, µm/s), straight-line velocity (VSL, µm/s), straightness (STR), and curvilinear velocity (VCL, µm/s), as well as progressive motility (%) and total motility (%), progressive motility slow (%), progressive motility circular (%), local motility (%), immotile (%). The analysis settings specific for bull spermatozoa were defined as follows: slow motility (VCL < 120.00 µm/s), local motility (VCL < 40.00 µm/s), immotile spermatozoa (HAC < 0.087), circular motility (radius > 10.00 µm; VSL < 10.00 µm/s), and definition of motile radius (<60.00 µm) with rotation > 0.70.

### Flow cytometry

Flow cytometric analysis was conducted using a FACSVerse™ cytometer (BD Biosciences, Becton Dickinson and Company, San Jose, CA, USA). Fluorescent dyes were excited by a 488 nm blue laser and a 405 nm violet laser. Emission signals were captured using specific band-pass filters: green fluorescence (FL1) at 527/32 nm, orange fluorescence (FL2) at 586/42 nm, red fluorescence (FL3) at 700/54 nm, and blue fluorescence (FL5) at 528/45 nm. Data generated from the sperm chromatin structure assay were processed using FCS Express 5 software (De Novo Software, Glendale, CA, USA).

#### Assessment of sperm membrane integrity, mitochondrial function, and chromatin integrity

Sperm quality parameters were assessed using flow cytometry, following the previously described methodology [14]. Plasma membrane integrity was evaluated using SYBR14 and propidium iodide (PI) stains (Live-Dead Sperm Viability Kit L-7011; Invitrogen, Eugene, OR, USA). Samples were adjusted to a concentration of 2 × 10^6^ sperm/mL in Buffer B (J.M. Morrell and H. Rodriguez-Martinez, patent pending). SYBR14 was diluted 1:50 in Buffer B, and 1.2 µL was added per sample, together with 3 µL of 2.4 mM PI. Following a 10-minute incubation at 38 °C, cells were classified as membrane-intact, membrane-damaged, or intermediate populations, of 10,000 events analysed.

Mitochondrial membrane potential was analyzed using tetraethylbenzimidazolylcarbocyanine iodide (JC-1) dye (3 mM stock), with 1.2 µL added to 300 µL of sperm sample (2.5 × 10^6^ cells/mL), incubated at 38 °C for at least 30 minutes. JC-1 aggregates (orange fluorescence, FL2) indicated high mitochondrial potential, while monomers (green fluorescence, FL1) indicated low potential. A total of 10,000 events were recorded per sample.

Chromatin integrity was assessed using the sperm chromatin structure assay (SCSA), based on acridine orange (AO) staining to measure DNA susceptibility to acid-induced denaturation. The DNA fragmentation index (%DFI) was calculated as the proportion of sperm with denatured single-stranded DNA (%DFI = red fluorescence/[green + red fluorescence] × 100). Samples were snap-frozen in Tris-sodium chloride and ethylene-diaminetetraacetic acid (TNE) buffer and stored at − 80 °C until analysis. Prior to staining, thawed samples were mixed with acid-detergent solution, followed by AO staining, and analyzed within 3–5 minutes.

### IVF

#### Oocyte collection and in vitro maturation

Bovine ovaries were collected at a local slaughterhouse and transported in warm (30 °C to 33 °C) saline solution (0.9% NaCl,) to the laboratory within 5 h. The COCs were aspirated from follicles sized 3–8 mm in diameter and collected in 25 mL tubes containing 75 μL heparin stock (5.000 IU/ml heparin). Evaluation of COCs was done in Wash-medium without heparin (Stroebech Media ApS, Hundested, Denmark). Groups of good acceptable quality COCs (grade I and II according to [18]) were selected, washed three times, and finally randomly divided into groups of 30 per well, in 4-well plates (Nunclon, 734-2693; Thermo Fisher Scientific, VWR Norway) containing 500 μL of IVM media (Stroebech Media ApS, Hundested, Denmark). The COCs were matured for 22 h at 38.5 °C under an atmosphere of 6% CO2 in air with maximum humidity.

#### IVF and culture

After maturation, the COCs were washed and transferred to new four-well plates containing IVF media (Stroebech Media ApS, Hundested, Denmark), and kept in the incubator (38.5 °C, 6% CO_2_, maximum humidity) while the semen samples were prepared. Semen samples were prepared in two steps, as described previously: first, curcumin treatment, and second, semen washing. In details, following the treatment, the 200 μL of each of the aliquots - treated and control - were first added to 3.8 mL of Sperm Wash, followed by centrifugation (330 ×g for 5 min). The supernatant was then removed, another 3.8 mL Sperm Wash were added, and the sample was centrifuged once more. After removing the supernatant after the second centrifugation, 6.8 mL of Sperm Wash were added and semen samples were centrifuged for the third time (330 ×g for 5 min). The supernatant was removed, and 300–500 µL Sperm Wash were added to the pellet. For each treatment and control semen pellet, post-washing sperm concentration was counted using a Burker chamber. Based on this concentration, an adjusted volume of spermatozoa suspension containing 0.25 × 10^6^ spermatozoa was added to each of the wells – curcumin treated spermatozoa in 2 wells and control spermatozoa in remaining 2 wells, in random order. The final volume in each well was 500 μL and final concentration of spermatozoa in each well was 0.5 × 10^6^ per mL for all wells. The 4-well fertilisation dish was incubated for 18–22 hours in 6% CO_2_, and maximum humidity at 38.5 °C. Following incubation, presumptive fertilized oocytes were first denuded by gentile pipetting and then washed and transferred to culture plates containing 500 μL IVC media (Stroebech Media ApS, Hundested, Denmark) with paraffin oil overlay (OvoilTM Vitrolife Sweden AB, Gothenburg, Sweden). Culture incubation was done in a humidified atmosphere of 6% O_2_, 6% CO_2_, and 78% N_2_ at 38.5 °C. At 44 h post-fertilization (pf), the cleavage rate was assessed, with the following rates recorded: numbers of non-cleaved oocytes, number of cleaved oocytes at 2-cell stage, number of cleaved oocytes at more than 2-cell stage. After further incubation of culture plates until day 8 after fertilization, the development stage was assessed again. Formed blastocysts at different stages were counted and their quality was evaluated according to the IETS guidelines. Different stages of blastocysts evaluated included: early blastocyst, blastocyst, expanded/expanding blastocyst, hatching/ hatched blastocyst.

Based on these records, the following rates were computed: cleavage rate counted as % of cleaved oocytes from initial group of COCs, and blastocyst rate counted as % of formed blastocysts from cleaved oocytes at day 2 post fertilization.

#### Staining nuclear count

Blastocysts collected on day 8 post-fertilization (pf) were subjected to staining and confocal microscopy to enable further evaluation. Nuclear staining was performed using DAPI (4′,6-diamidino-2-phenylindole; ThermoFisher Scientific, USA) to visualize cell nuclei. Embryos were fixed in 2% paraformaldehyde in PBS for 30 minutes at room temperature, followed by rinsing in PBS. Embryos were mounted on microscope slides using antifade mounting medium with DAPI (Vectashield, Vector Laboratories) and examined under a fluorescence microscope. The number of nuclei were assessed and recorded to evaluate number of blastomeres.

### Statistical analysis

Statistical analyses were performed using R software (version 4.4.2.). To assess the effect of curcumin-treated bull semen on IVF outcomes, linear mixed-effects models (LMMs) was used, with treatment (Control vs. Curcumin) as a fixed effect and batch included as a random effect to account for repeated measures. Dependent variables analysed included the total number of COCs in maturation, percentage (%) of cleavage rate, percentage (%) of oocytes with more than 2 cells from cleaved, and percentage (%) of blastocyst rate on day 8. Assumptions of normality and homoscedasticity were assessed via residual diagnostics. In addition, to assess sperm quality parameters between control and treatment group, paired t-tests were applied. The results are presented as Mean and standard deviation (SD). The significance threshold was set at *p* < 0.05.

## Results

There were no significant differences between the Curcumin-treated and Control groups for any of the evaluated IVF parameters. The total number of COCs in maturation for Curcumin were 311 ± 2.55 and for Control 317 ± 1.79.

**Fig. 2:**
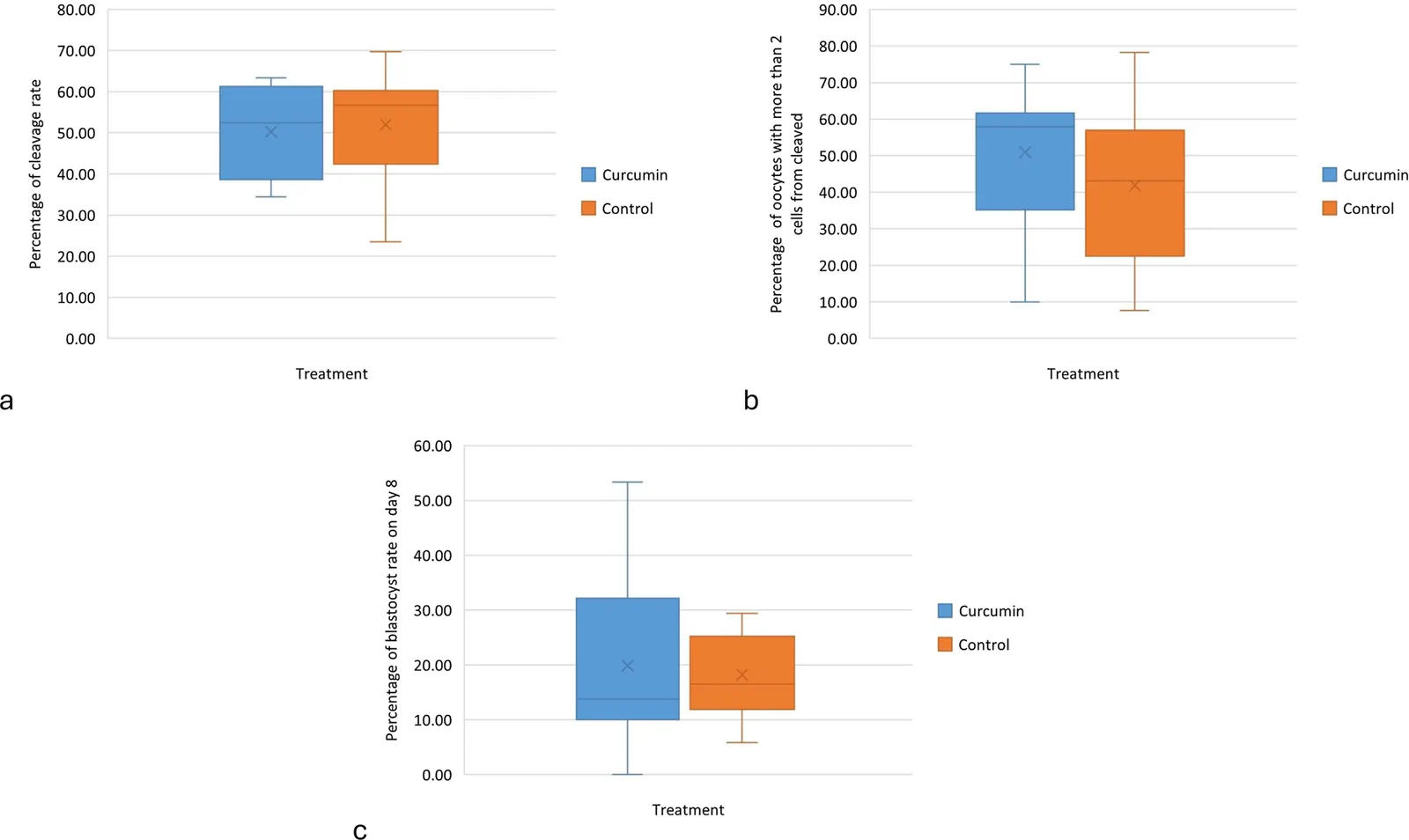
In vitro embryo development outcomes: **a** - percentage of cleaved oocytes between control and curcumin treated bull semen. **b** - percentage of cleaved oocytes above the 2-cell stage between control and curcumin treated bull semen. **c** - proportion of blastocysts day 8 post fertilization between control and curcumin treated bull semen

Cleavage rates are presented in Fig. 2a and did not differ significantly between control (51.97±13.58) and treatment (50.25±10.89), (Estimate = −1.722, SE = 5.233, *p* = 0.747).

Similar findings were observed for Percentage of oocytes with >2 cells from cleaved (control 41.88 ± 22.18 vs treatment 50.94 ± 19.46, Estimate = 9.051, SE = 5.980, *p* = 0.15) and blastocyst rate on day 8 (control 18.23 ± 7.80 vs treatment 19.87 ± 15.77, Estimate = 1.642, SE = 3.643, *p* = 0.65) presented in Fig. 2b and c.

The treatment did not have a significant effect on sperm motility and kinematic parameters. However, a trend towards significance was observed for total motility and immotile spermatozoa, with these parameters being higher in the Curcumin-treated group compared to the control group (Table 1).

**Table 1:** Sperm motility and kinematic parameters between control and curcumin treated bull semen

	Control	Curcumin
Total motility (%)	68.33 ± 3.03^T^	73.51 ± 4.35^T^
Progressive motility (%)	67.48 ± 3.11	72.59 ± 4.48
Progressive motility - slow (%)	51.67 ± 4.60	55.45 ± 8.76
Progressive motility - circular (%)	14.99 ± 2.67	16.20 ± 6.08
Local motility (%)	0.85 ± 0.32	0.92 ± 0.32
Immotile (%)	31.67 ± 3.03^T^	26.48 ± 4.35^T^
VCL (µm/s)	122.00 ± 9.96	144.39 ± 23.96
VSL (µm/s)	51.26 ± 4.80	55.30 ± 7.38
VAP (µm/s)	61.04 ± 5.20	65.78 ± 8.46
ALH (µm)	1.49 ± 0.11	1.57 ± 0.13
BCF (Hz)	11.95 ± 0.77	12.26 ± 0.67
WOB (VAP/VCL)	0.50 ± 0.01	0.44 ± 0.11
LIN (VSL/VCL)	0.42 ± 0.01	0.42 ± 0.01
STR (VSL/VAP)	0.84 ± 0.02	0.84 ± 0.01

Abbreviation: VCL—curvilinear velocity, VAP—average path velocity, VSL—straight line velocity, LIN—linearity, STR—straightness, WOB—wobble, BCF—beat cross frequency, ALH—amplitude of lateral head displacement; Superscript letters within a row denote trend toward significant ^T^*p* = 0.09

The viability parameters remained similar between the control and curcumin-treated groups for the majority of sperm quality parameters. Nonetheless, mitochondrial membrane potential (MMP) was significantly lower in the curcumin-treated semen compared to the control group (Table 2).

**Table 2:** Sperm viability parameters of control and curcumin treated sperm evaluated by Flow cytometry

	Control	Curcumin
MI live (%)	63.63 ± 2.42	63.49 ± 1.59
MI dying (%)	36.20 ± 2.43	36.37 ± 1.59
MI dead (%)	0.17 ± 0.02	0.14 ± 0.05
MMP high (%)	25.52 ± 7.20^a^	9.47 ± 3.90^a^
MMP low (%)	74.48 ± 7.20^a^	90.33 ± 3.90^a^
%DFI	2.06 ± 0.48	2.18 ± 0.58

Abbreviation: MI—Membrane integrity, MMP—Mitochondrial membrane potential, DFI—DNA fragmentation index; Superscript letters within a row denote significant differences compared with control: a—*p* < 0.001

The number of nuclei was assessed to evaluate embryo cell count on Day 8 post-fertilization. There were no significant differences observed between the control and curcumin-treated groups. Similarly, the mean number of blastomeres was 74 ± 26 in the control group and 72 ± 22 in the treated group.

## Discussion

The objective of this study was to evaluate whether curcumin treatment of sperm affects their in vitro fertilizing capacity. The results indicated that curcumin treatment did not significantly affect the in vitro fertilization (IVF) outcomes, as no statistically significant differences were observed in the cleavage rate, the percentage of oocytes with more than two cells, or the percentage of blastocysts on day 8 between the curcumin-treated group and the control group. These findings suggest that curcumin did not negatively impact the fertilization capacity of bull sperm, at least under the experimental conditions used in this study. It is worth noting that the observed cleavage and blastocyst rates in both the control and curcumin-treated groups were markedly lower than those typically reported in standard IVF protocols, where approximately 50% of cleaved oocytes are expected to develop into blastocysts [19]. The lower embryo development observed in the present study should be interpreted with caution, as multiple factors may contribute to variation in embryo developmental competence. Although the spermatozoa concentration used for IVF was sufficient to support fertilization, the use of a reduced sperm concentration may represent one possible factor influencing subsequent embryo development. In our study, a concentration of 0.5 × 10^6^ spermatozoa/mL was used, which is half of the 1 × 10^6^ spermatozoa/mL recommended by the media manufacturer. This intentional reduction aimed to enhance the visibility of any effects associated with curcumin treatment, avoiding potential masking because of an excessively high sperm concentration [17, 20]. Additionally, both control and treated sperm samples were incubated at room temperature for 30 minutes prior to fertilization, a factor that may have negatively influenced sperm fertilizing capacity (by affecting sperm quality) and subsequent embryo development. However, an important additional source of variability is the use of ovaries collected from slaughterhouse-derived animals. Unlike oocytes obtained from donors with a controlled hormonal environment, slaughterhouse-derived ovaries originate from animals with potentially different reproductive, hormonal, nutritional and physiological backgrounds [21]. Finally, differences in the stress experienced before slaughter and during transport may influence follicular and oocyte quality. These factors can affect oocyte maturation, developmental competence and subsequent embryo development, and they cannot be completely controlled under slaughterhouse conditions [22]. Therefore, differences observed in embryo development should not be attributed solely to sperm concentration; variation in the intrinsic quality of the oocytes and their developmental competence may also have contributed to the observed outcomes. It should be noted that, under consistent culture conditions and using the same media, our laboratory typically observes a cleavage rate about 80% and a blastocyst formation rate ranging from 20% to 50%, calculated based on the number of oocytes in maturation. Furthermore, the positive value of the estimate for curcumin treatment suggests that curcumin may lead to better outcome for the assessed parameters, including the cleavage rate and the percentage of blastocysts on day 8, although no statistical significance was observed. Interestingly, sperm quality analyses showed a trend toward significance for total motility, which was higher in the curcumin-treated group, and the proportion of immotile sperm, which was lower in the curcumin-treated group. These findings suggest that curcumin may have a beneficial effect on sperm motility, which aligns with previous studies reporting antioxidant properties of curcumin that help to reduce oxidative stress and enhance sperm function [9, 23]. The observed trend towards improved motility might be attributed to curcumin’s ability to reduce oxidative damage to sperm cells, which is known to compromise motility and overall sperm function.

The mitochondrial membrane potential (MMP) results indicated a significant difference between the groups. The proportion of sperm with high MMP was lower in the curcumin-treated group, which may indicate a potential negative effect of curcumin on mitochondrial function. Mitochondria play a crucial role in sperm energy production, and a reduction in MMP could lead to compromised sperm bioenergetics, potentially impacting fertilization efficiency. A possible explanation for this effect could be curcumin’s interaction with mitochondrial electron transport chain components, leading to altered mitochondrial function [24, 25]. Some studies have suggested that curcumin, at higher concentrations, may have pro-oxidant effects, which could contribute to mitochondrial dysfunction [26]. This action on mitochondria of spermatozoa has not been evaluated yet. Additionally, the observed reduction in MMP from the beginning of the experiment suggests that factors other than curcumin treatment may have influenced mitochondrial activity. Cryopreservation itself is known to induce mitochondrial damage due to the generation of reactive oxygen species (ROS) and disruption of the mitochondrial membrane [27]. Since the sperm samples were cryopreserved before the experiment, it is possible that pre-existing mitochondrial alterations were present, which were exacerbated by curcumin treatment.

When comparing our findings to previous studies, the impact of curcumin on sperm function remains controversial. Some studies have reported positive effects of curcumin on sperm motility and viability [28], while others have demonstrated detrimental effects at higher concentrations [23]. The concentration and exposure time of curcumin play a crucial role in determining its effects, and further studies are required to optimize these parameters for application in semen extenders. Previous studies have reported improvements in mitochondrial membrane potential (MMP) when fresh semen was treated with curcumin prior to cryopreservation, with the beneficial effects observed after thawing [28]. This suggests that curcumin may have protective properties against cryodamage, potentially by reducing oxidative stress and maintaining mitochondrial function. Interestingly, in the present study, where semen was treated with curcumin post-thawing, the observed MMP was lower in the treated group. This difference may be due to differences in the timing of curcumin exposure, as pre-cryopreservation treatment may provide a protective effect against freezing-induced mitochondrial damage, while post-thaw treatment could interact differently with already-damaged mitochondria. Another factor contributing to these differences could be individual bull variation in sperm quality and cryotolerance, which is known to impact post-thaw sperm viability and function [29,30 ]. Additionally, variations in plant-based substances, such as differences in curcumin source, concentration, and bioavailability, can also influence the outcomes of sperm treatment [12]. An important consideration for the future application of curcumin in semen preservation is its low aqueous solubility and limited bioavailability, which may restrict its biological effectiveness and make its activity dependent on the formulation and vehicle used for delivery. Consequently, nanotechnology-based formulations have been investigated as a strategy to improve the solubility, stability, and bioavailability of curcumin. In reproductive research, curcumin nanoparticles have shown promising effects on sperm preservation. For example, supplementation with curcumin nanoparticles has been reported to improve post-thaw sperm quality compared with conventional curcumin in rabbit spermatozoa [31], while a curcumin oil nano-emulsion improved several sperm quality, oxidative stress, and fertilizing-capacity parameters in cryopreserved buffalo sperm [13]. More recently, a nanoliposomal curcumin formulation improved post-thaw motility, viability, membrane and acrosome integrity, and oxidative status and was associated with improved fertility outcomes in cryopreserved ram sperm [32]. In addition, recent evidence in rabbit buck sperm indicates that the biological effects of curcumin nanoparticles may depend on particle size, with smaller nanoparticles showing more favourable effects on sperm motility and DNA integrity, whereas larger particles were associated with reduced mitochondrial membrane potential [33]. Taken together, these findings suggest that future studies should not only evaluate curcumin concentration but also consider formulation, delivery system, particle size, and dose when developing curcumin-based additives for semen preservation and fertility applications.

The observed decrease in MMP following curcumin treatment in already cryopreserved samples may explain why no significant differences in IVF results were observed between groups. It is possible that curcumin’s effects on mitochondrial function counteracted any potential benefits on motility or sperm viability, ultimately leading to similar fertilization outcomes. Future studies should assess whether treating fresh semen with curcumin before IVF would yield improved fertilization results, potentially overcoming the mitochondrial dysfunction observed in cryopreserved samples.

A limitation of the present study is the use of semen from a single proven bull, which may limit the generalizability of the findings because bull-to-bull variation in sperm cryotolerance, mitochondrial function, antioxidant responses, and fertilizing capacity may influence the response to curcumin supplementation [34]. The use of a single bull was intentional and based on the availability of semen from a bull with previously characterized fertility in our IVF system, thereby allowing the effect of curcumin supplementation on in vitro fertilization outcomes to be evaluated under controlled conditions while minimizing variation associated with differences in inherent fertility among bulls in IVF. Overall, while curcumin showed a trend towards improving sperm motility, its effect on mitochondrial function warrants further investigation. Future studies should explore the mechanisms underlying curcumin’s impact on sperm bioenergetics, test different concentrations and exposure times, and assess long-term effects on embryo development. Understanding these factors will be essential for determining the feasibility of curcumin as an alternative to antibiotics in semen extenders while ensuring optimal sperm function and fertilization potential. The use of a single concentration represents a limitation of the study and does not allow evaluation of dose-dependent effects. However, this concentration was chosen as a biologically relevant and previously validated dose [14]. Given the absence of detrimental effects in the present study, future experiments should evaluate the effects of curcumin supplementation before freezing to determine its potential role during cryopreservation.

Recent studies explored the potential of curcumin as an antioxidant additive in reproductive technologies. For example, Lin et al. [15] reported improved fertilization and blastocyst development rates when curcumin was added to both semen extenders and oocyte in vitro maturation (IVM) media at concentrations of 25–50 µM. Their findings also included enhanced mitochondrial membrane potential (MMP) and antioxidant enzyme activity in both sperm and oocytes. In contrast, a key difference between their approach and the present study lies in the timing and dosage of curcumin exposure. While applied curcumin prior to cryopreservation and during oocyte maturation [15], we assessed the effects of a 5% curcumin extract applied to semen post-thaw, under IVF conditions. Notably, we observed no significant differences in fertilization or embryo development rates between curcumin-treated and control groups. Furthermore, in contrast to the mitochondrial enhancement reported by [15], our results showed a significant reduction in MMP following curcumin treatment. These findings suggest that the effects of curcumin on sperm function and fertilization potential may be highly dependent on both treatment timing and concentration, as well as the physiological state of the sperm (i.e., pre- vs. post-cryopreservation). Further studies are needed to clarify these interactions and optimize curcumin use in semen preservation protocols.

The decision to evaluate curcumin treatment on spermatozoa after thawing was based on both practical considerations and biological rationale. Cryopreservation is known to cause oxidative stress, membrane disruption, and mitochondrial damage in spermatozoa, with many of these injuries manifesting during the thawing process. Applying curcumin post-thaw offers a targeted opportunity to mitigate this secondary oxidative damage, particularly during the handling and fertilization stages. Furthermore, this approach aligns with real-world reproductive practices, where modifying thawed semen prior to artificial insemination may be more feasible than altering standard cryopreservation protocols. Despite the widespread interest in curcumin as an antioxidant, relatively few studies have examined its effects when applied post-thaw, making this investigation both timely and novel.

## Conclusion

The present study demonstrates that curcumin supplementation in thawed semen does not adversely affect fertilization outcomes under in vitro fertilization (IVF) conditions, as no significant differences were observed between curcumin-treated and control groups. However, the observed reduction in mitochondrial membrane potential (MMP) following curcumin supplementation indicates an alteration in sperm mitochondrial status, despite the maintenance of fertilization capacity under the conditions evaluated. These findings suggest that curcumin supplementation at the tested concentration does not impair the fertilizing ability of thawed spermatozoa, although its effects on sperm mitochondrial function warrant further investigation. Given these findings, further research is warranted to evaluate the efficacy and safety of curcumin-treated semen in vivo, including its effects on pregnancy rates, embryo development, and offspring health following artificial insemination with curcumin-treated, cryopreserved semen.

## Data Availability

No datasets were generated or analysed during the current study.
